# Design of Shape Forming Elements for Architected Composites via Bayesian Optimization and Genetic Algorithms: A Concept Evaluation

**DOI:** 10.3390/ma17215339

**Published:** 2024-10-31

**Authors:** David O. Kazmer, Rebecca H. Olanrewaju, David C. Elbert, Thao D. Nguyen

**Affiliations:** 1 Department of Plastics Engineering, University of Massachusetts Lowell, Lowell, MA 01854, USA; rebecca_olanrewaju@student.uml.edu; 2 Department of Mechanical Engineering, Johns Hopkins University, Baltimore, MD 21218, USA; elbert@jhu.edu (D.C.E.); vicky.nguyen@jhu.edu (T.D.N.)

**Keywords:** architected composites, additive manufacturing, artificial intelligence, optimization

## Abstract

This article presents the first use of shape forming elements (SFEs) to produce architected composites from multiple materials in an extrusion process. Each SFE contains a matrix of flow channels connecting input and output ports, where materials are routed between corresponding ports. The mathematical operations of rotation and shifting are described, and design automation is explored using Bayesian optimization and genetic algorithms to select fifty or more parameters for minimizing two objective functions. The first objective aims to match a target cross-section by minimizing the pixel-by-pixel error, which is weighted with the structural similarity index (SSIM). The second objective seeks to maximize information content by minimizing the SSIM relative to a white image. Satisfactory designs are achieved with better objective function values observed in rectangular rather than square flow channels. Validation extrusion of modeling clay demonstrates that while SFEs impose complex material transformations, they do not achieve the material distributions predicted by the digital model. Using the SSIM for results comparison, initial stages yielded SSIM values near 0.8 between design and simulation, indicating a good initial match. However, the control of material processing tended to decline with successive SFE processing with the SSIM of the extruded output dropping to 0.023 relative to the design intent. Flow simulations more closely replicated the observed structures with SSIM values around 0.4 but also failed to predict the intended cross-sections. The evaluation highlights the need for advanced modeling techniques to enhance the predictive accuracy and functionality of SFEs for biomedical, energy storage, and structural applications.

## 1. Introduction

Recent advances in modeling, simulation, and manufacturing are accelerating the discovery of new materials while also reducing the time and cost of bringing new products to market. The materials genome initiative [1,2] reinforces these trends by seeking to accelerate materials discovery to enable materials-by-design wherein material and ultimate product properties are predicted from first principles; the end goal is to benefit humanity by creating new materials that support novel applications while reducing cost and environmental impact. Architected and cellular material composites offer promising avenues for creating efficient structures with previously impossible properties. Examples include lightweight structures and “white-space” materials [3] with gradient properties [4], negative Poisson’s ratio [5], and other tunable properties [6]. While nature provides abundant examples of architected materials [7], a review of the literature suggests that most of the recent work relies on additive manufacturing methods to achieve complex material placement at the mesoscale and microscale. Kladovasilakis et al. [8] provide a recent review with a classification of architected materials based on the geometry, e.g., stochastic, homogeneous, and pseudo-periodic architectures. There are at least two issues with the use of additive manufacturing (AM) as the production route for architected materials. First, while layer-based AM processes such as Continuous Liquid Interface Production (CLIP) [9] and Computed Axial Lithography (CAL) [10] are substantially faster than extrusion-based and laser-based processes, they remain extremely slow and energy intensive relative to net-shape manufacturing processes [11,12]. Second, products made by AM processes have significant failure modes related to finite layer thickness [13], fatigue [14], and quality assurance [15]. Together, these issues preclude the adoption of architected materials in mass production with the most prevalent applications being lattice-based structural designs with mesoscale features [16].

This article provides the first investigation of the design and use of shape forming elements as a method for the mass production of architected material composites. The goal is to provide a structured methodology for designing architected composites that supports efficient and scalable production. The approach is a two-dimensional extension of one-dimensional layer multiplying elements [17] used in polymer processing to create multi-layered sheets [18,19], tubes [20,21], and simple hierarchical structures [22]. As described, a shape forming element (SFE) converts an input cross-section to an output cross-section with differing geometry through the use of multiple flow channels that deform, rotate, and shift the local distributions of materials; multiple SFE stages are then used to achieve higher levels of complexity of architected composites with high production rates when implemented in processes such as extrusion and injection molding. The article then describes implementations of design automation using Bayesian optimization and generic algorithms. Validation of the SFE designs using flow simulation and rapid prototyping is described, and significant implementation issues with the SFE concept related to optimization of the architected composites and methods of manufacture are identified. Accordingly, suggested modeling extensions are discussed to enable broad and practical applications in various industries, including biomedical and aerospace.

## 2. Materials and Methods

### 2.1. Shape Forming Elements

**Figure 1:**
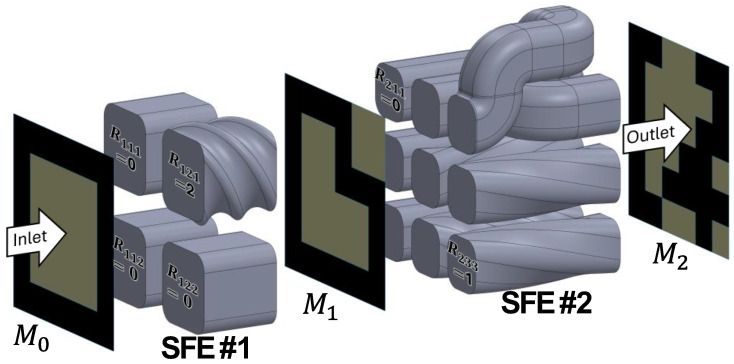
Flow channel designs implemented in a first SFE with the rotation matrix of Equation (3) and a second SFE with a rotation matrix of Equation (6) also with a shift matrix of Equation (8). The material traverses from left to right resulting in the three material cross-sections *M*_0_, *M*_1_, and *M*_2_ corresponding to Equations (1), (5) and (9).

Consider a cross-section composed of two materials processed through sets of flow channels provided within two shape forming elements (SFEs) as shown in Figure 1. There are at least five operations that each SFE can provide: (1) cutting, (2) rotation, (3) shifting, (4) combining, and (5) reshaping. The first four operations are the focus of this article; reshaping SFEs are in concurrent development but in need of further research with respect to design automation. Other SFE functions such as flipping, copying, and deleting are easy to model in theory but difficult to realize in practice without transient actions implemented within the manufacturing process.

The input section of Figure 1, $M_{0}$, is modeled as two materials in a 6 × 6 matrix:

(1) $$M_{0}=\left[\begin{array}{cccccc} 1 & 1 & 1 & 1 & 1 & 1\\ 1 & 0 & 0 & 0 & 0 & 1\\ 1 & 0 & 0 & 0 & 0 & 1\\ 1 & 0 & 0 & 0 & 0 & 1\\ 1 & 0 & 0 & 0 & 0 & 1\\ 1 & 1 & 1 & 1 & 1 & 1 \end{array}\right]$$

The use of the {0,1} material representation allows for straightforward computation of the relative material concentrations. For the defined $M_{0}$, material 1 is located at 20 of 36 overall locations, so the concentration of material 1 is 0.55. While a 6 × 6 matrix is used for illustration purposes, larger matrices are later analyzed for providing improved resolution across multiple shape forming operations. Multiple materials and their blends can be modeled by extension.

A rotation matrix for each i-th SFE, $R_{ijk}$, is defined for each *jk*-th port representing a partition of the input material matrix *M_i_*. The number and locations of the ports define the number of cuts and partitions, $P_{jk}$, of the input material matrix. For example, suppose that a first SFE has a 2 × 2 set of ports, as shown in Figure 1. Then, $M_{0}$ can be represented as a set of $P_{jk}$ as

(2) $$M_{0}=\left[\begin{array}{cc} P_{11}=\left[\begin{array}{ccc} 1 & 1 & 1\\ 1 & 0 & 0\\ 1 & 0 & 0 \end{array}\right] & P_{12}=\left[\begin{array}{ccc} 1 & 1 & 1\\ 0 & 0 & 1\\ 0 & 0 & 1 \end{array}\right]\\ P_{21}=\left[\begin{array}{ccc} 1 & 0 & 0\\ 1 & 0 & 0\\ 1 & 1 & 1 \end{array}\right] & P_{22}=\left[\begin{array}{ccc} 0 & 0 & 1\\ 0 & 0 & 1\\ 1 & 1 & 1 \end{array}\right] \end{array}\right]$$

The material processed through each *jk*-th port can be rotated relative to the center-line axis of the swept section in its flow direction. For the example of Figure 1 in which $R_{121}=$ 2, a rotation matrix in which only the top right port is rotated 180° in the first SFE is:

(3) $$R_{1jk}=\frac{\pi }{2}\left[\begin{array}{cc} 0 & 2\\ 0 & 0 \end{array}\right],$$

where in the output $M_{i,rot}$ from the rotation function, *rot*(), for the *i*-th SFE is:

(4) $$M_{i,rot}=rot\left(M_{i-1},R_{i}\right)=\left[\left[rot90\left(P_{ijk},R_{ijk}\right)\right]\right].$$

Here, the *rot*90() operation is the built-in Matlab (Mathworks, Waltham, MA, USA) function for rotating a matrix by 90° counterclockwise $R_{jk}$ times, allowing for the straightforward manipulation of matrix orientations. An interactive design function *Design_SFE*() is provided in the Supplementary Materials along with other routines for optimization as later described. Appendix C provides a synopsis of these and other Matlab built-in and developed functions used in the performance of the research; all developed functions are available as described in the Supplementary Materials Section.

The result of Equations (2)–(4) is the first output material, $M_{1}$:

(5) $$M_{1}=\left[\begin{array}{cc} P_{111,rot}=\left[\begin{array}{ccc} 1 & 1 & 1\\ 1 & 0 & 0\\ 1 & 0 & 0 \end{array}\right] & P_{112,rot}=\left[\begin{array}{ccc} 1 & 0 & 0\\ 1 & 0 & 0\\ 1 & 1 & 1 \end{array}\right]\\ P_{121,rot}=\left[\begin{array}{ccc} 1 & 0 & 0\\ 1 & 0 & 0\\ 1 & 1 & 1 \end{array}\right] & P_{122,rot}=\left[\begin{array}{ccc} 0 & 0 & 1\\ 0 & 0 & 1\\ 1 & 1 & 1 \end{array}\right] \end{array}\right]$$

Successive rotation and shifting operations may be defined and applied. For example, suppose that material $M_{1}$ is fed into a second SFE with a set of 3 × 3 ports and a rotation matrix defined as:

(6) $$R_{2}=\frac{\pi }{2}\left[\begin{array}{ccc} 0 & 0 & 0\\ 0 & 3 & 1\\ 0 & 2 & 1 \end{array}\right]$$

Then, $M_{2}=rot\left(M_{1,rot},R_{2}\right)$ would be

(7) $$M_{2}=\left[\begin{array}{ccc} rot90\left(\left[\begin{array}{cc} 1 & 1\\ 1 & 0 \end{array}\right],0\right) & rot90\left(\left[\begin{array}{cc} 1 & 1\\ 0 & 1 \end{array}\right],0\right) & rot90\left(\left[\begin{array}{cc} 0 & 0\\ 0 & 0 \end{array}\right],0\right)\\ rot90\left(\left[\begin{array}{cc} 1 & 0\\ 1 & 0 \end{array}\right],0\right) & rot90\left(\left[\begin{array}{cc} 0 & 1\\ 0 & 0 \end{array}\right],3\right) & rot90\left(\left[\begin{array}{cc} 1 & 1\\ 0 & 1 \end{array}\right],1\right)\\ rot90\left(\left[\begin{array}{cc} 1 & 0\\ 1 & 1 \end{array}\right],0\right) & rot90\left(\left[\begin{array}{cc} 0 & 0\\ 1 & 1 \end{array}\right],2\right) & rot90\left(\left[\begin{array}{cc} 0 & 1\\ 1 & 1 \end{array}\right],1\right) \end{array}\right]=\left[\begin{array}{cccccc} 1 & 1 & 1 & 1 & 0 & 0\\ 1 & 0 & 0 & 1 & 0 & 0\\ 1 & 0 & 0 & 0 & 1 & 1\\ 1 & 0 & 0 & 1 & 1 & 0\\ 1 & 0 & 1 & 1 & 1 & 1\\ 1 & 1 & 0 & 0 & 1 & 0 \end{array}\right]$$

Each SFE can also shift the material flows from the input port locations to different output port locations using defined shift matrices, $S_{X,ijk}$ and $S_{Y,ijk}$, in which integer elements represent the relative movement between the *jk*-th ports located along the respective *X* and *Y* axes in the *i*-th SFE. For example, the second SFE of Figure 1 swaps the top center and top right port outputs by defining $S_{X,2jk}\;$ as:

(8) $$S_{X,2jk}=\left[\begin{array}{ccc} 0 & 1 & -1\\ 0 & 0 & 0\\ 0 & 0 & 0 \end{array}\right]$$

Applying the *shift*() function to the shift matrix of Equation (8) with the material distribution of Equation (7) yields:

(9) $$M_{2,shift}=shift\left(M_{2,rot},S_{X,2jk}\right)=\left[\begin{array}{cccccc} 1 & 1 & 0 & 0 & 1 & 1\\ 1 & 0 & 0 & 0 & 0 & 1\\ 1 & 0 & 0 & 0 & 1 & 1\\ 1 & 0 & 0 & 1 & 1 & 0\\ 1 & 0 & 1 & 1 & 1 & 1\\ 1 & 1 & 0 & 0 & 1 & 0 \end{array}\right]$$

Figure 1 depicts the flow channels of the first and second SFE examples along with the modeled architected composite cross-sections wherein the lighter and darker sections, respectively, represent materials 0 and 1.

### 2.2. Design Automation

A salient feature of the shape forming element method is that many designs can be achieved across multiple SFE stages. However, achieving a target architecture by manual design is literally puzzling, akin to a tiling puzzle [23], with non-obvious solutions. A significant issue is that the rotation of the material partitions can be hard to visualize across SFE stages to achieve desired material architectures. Furthermore, the number of combinations that may be considered is staggering. The number of combinations is on the order of $d^{p}$ where *p* represents the number of design parameters and *d* represents the number of degrees of freedom for each design parameter (typically four considering only port rotation but much greater when shifting and other transforms are considered). The later design examples have a design space comprising around 4^55^ or 1.27 $\times \;10^{30}$ combinations. Thus, design automation was implemented using Bayesian optimization and genetic algorithms.

Arróyave and McDowell [24] provide a review of systems approaches for materials design in which they suggest that “the most efficient methods for materials discovery are based on variants of Bayesian optimization (BO)”. According to Brochu [25], “Bayesian optimization employs the Bayesian technique of setting a prior over the objective function and combining it with evidence to get a posterior function. This permits a utility-based selection of the next observation to make on the objective function, which must take into account both exploration (sampling from areas of high uncertainty) and exploitation (sampling areas likely to offer improvement over the current best observation)”. Thus, Bayesian optimization allows for the exploration of a vast design space more efficiently than traditional methods, which is particularly advantageous in the context of complex material architectures where intuitive design approaches might fail.

**Figure 2:**
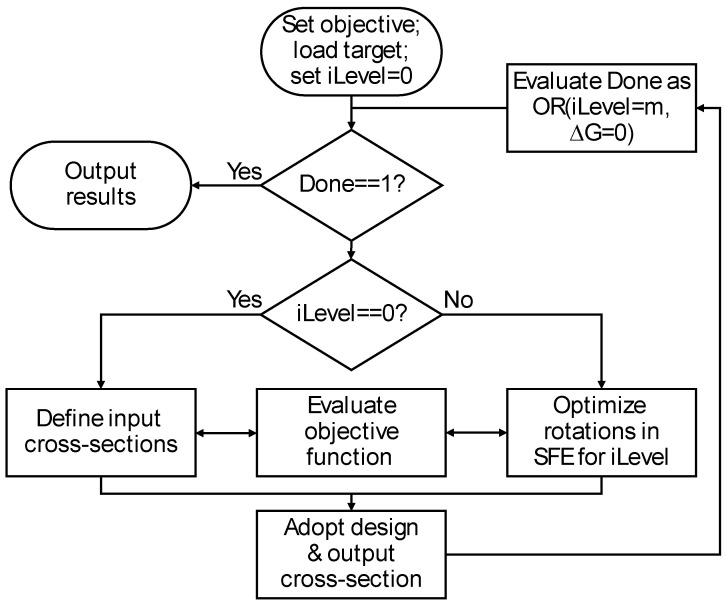
Implemented algorithm for shape function element (SFE) design using sequential Bayesian optimization.

Striving for intelligent design through design automation, integer optimization was first implemented using the Matlab function *bayesopt*() to design a series of SFEs to minimize an objective function G according to the flowchart of Figure 2. The algorithm iteratively performs successive levels of design optimization, starting with the initial level (iLevel = 0). In this initial level, the algorithm loops through multiple input cross-sections (later demonstrated) and evaluates the objective function for each and selecting the best. The Bayesian optimization then progresses for each SFE, allowing the algorithm to refine and optimize the number and specific rotation of the flow channels to minimize the objective value. After each iteration, the selected design and resulting output section are adopted for subsequent analysis until the number of SFE stages is met (iLevel = m) or there is no improvement in the objective function (ΔG = 0).

To assist the evolution of the architected cross-section across sequential SFE and Bayesian optimization stages, a first objective function $G_{1}$ was developed so that the output material matches a target cross-section design represented as a binary image, $I_{Target}$. Mathematically, this first objective is achieved by minimizing $G_{1}$, which is defined as:

(10) $$G_{1}=w\cdot MSSE\left(I_{Target},I_{Output}\right)+\left(1-w\right)\left(1-SSIM\right)\left(I_{Target},I_{Output}\right)$$

Here, MSSE represents the mean sum squared error representing a pixel-by-pixel comparison of the target image, $I_{Target}$, and the output image, $I_{Output}$, computed as:

(11) $$MSSE\left(I_{Target},I_{Output}\right)=\sum {\left(I_{Target}-I_{Output}\right)}^{2}/N^{2},$$

where the two images are binary (as in the representation of Equation (1) and scaled to size N; this definition is similar to the Matlab function *immse*(). Meanwhile, SSIM represents the structural similarity index for measuring image quality as defined by Wang et al. [26]. Since SSIM approaches a value of 1 with perfect correlation, the value of (1 − SSIM) is calculated as the error norm in the objective function for minimization purposes. The purpose of the scalar weighting value, w, is to balance between the trade-off between local pixel matching driven by MSSE and overall structural similarity driven by SSIM. A value of 0.5 was typically used as the weighting value with similar execution time and results for non-extreme values, e.g., $w\in \left(0.1,0.9\right)$.

**Figure 3:**
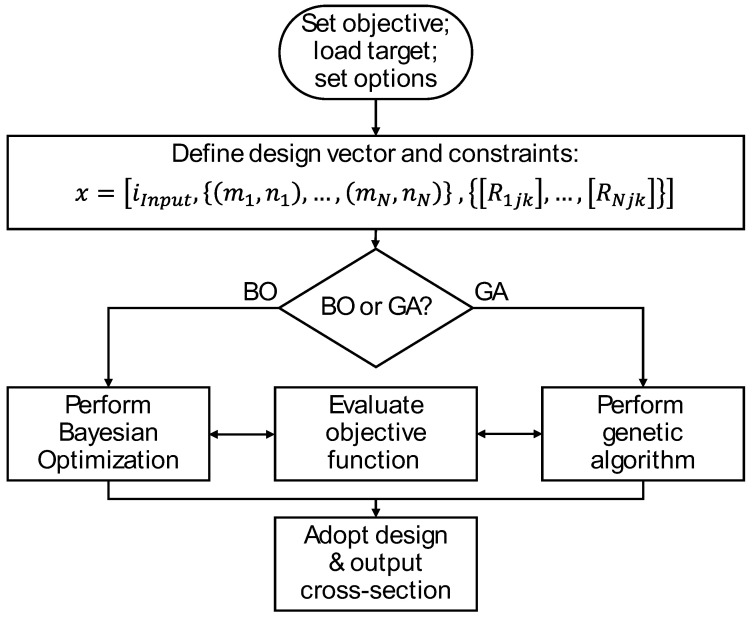
Implemented algorithm for shape function element (SFE) design using concurrent Bayesian optimization or genetic algorithms.

As later characterized, the testing and validation of Bayesian optimization using the sequential algorithm of Figure 2 frequently resulted in poor results. The reason was that the optimal design requires the selection of input cross-section and early SFE stages that are far from optimal as measured by the objective function. In other words, the sequential Bayesian optimization falls prey to the same limitation of human sequential design thinking in which the architected design only comes together in the latter SFE stages based on unobvious intermediate cross-sections. As such, a different integer optimization was implemented according to the flowchart of Figure 3 in which all design parameters are concurrently optimized. The vector of design parameters is defined as:

(12) $$x=\left[i_{Input},\left\{\left(m_{1},n_{1}\right),\ldots ,\left(m_{N},n_{N}\right)\right\},\left\{\left[R_{1jk}\right],\ldots ,\left[R_{Njk}\right]\right\}\right],$$

where $R_{ijk}$ is the rotation matrix for a given stage i per Equation (3), $m_{i}$ and $n_{i}$ are the number of SFE ports in the *x* and *y* directions, and $i_{Input}$ is an integer representing the input material cross-section. This implementation allows for non-square fluid ports, although square ports are also implemented with the assumption that $m_{i}=n_{i}$ and a corresponding reduction in the length of the design vector x.

Given this problem structure, it was straightforward to also implement a solution based on genetic algorithms (GAs) as shown in the flowchart of Figure 3. This implementation calls Matlab’s *ga*() function that mimics the process of natural selection to iteratively improve a population of candidate solutions [27]. The GA’s convergence is guided by constrained optimization as described by Conn, Gould, and Toint in their works on globally convergent augmented Lagrangian algorithms [28]. The resulting GA method is generally well adapted to handling large systems with complex constraints. Subsequent testing showed that GA tended to outperform BO, which tended to converge to local, suboptimal solutions.

The first objective $G_{1}$ per Equation (10) was developed for SFE design automation to match a target image. For demonstration purposes, a second objective, $G_{2}$, is defined as:

(13) $$G_{2}=SSIM\left(I_{White},I_{Output}\right)$$

Here, $I_{White}$ represents a plain white image such that the intent is to maximize the difference between the output and a uniform material. In other words, the minimization of $G_{2}$ should drive the SFEs to produce a highly complex, architected material like a T-square or other fractal [29] as related to the concepts of negentropy [30] and Gibbs’ free energy [31]. While the objectives $G_{1}$ and $G_{2}$ are used as examples, other objective functions are needed to derive architectures to maximize material and product performance.

### 2.3. Physical Prototyping

**Figure 4:**
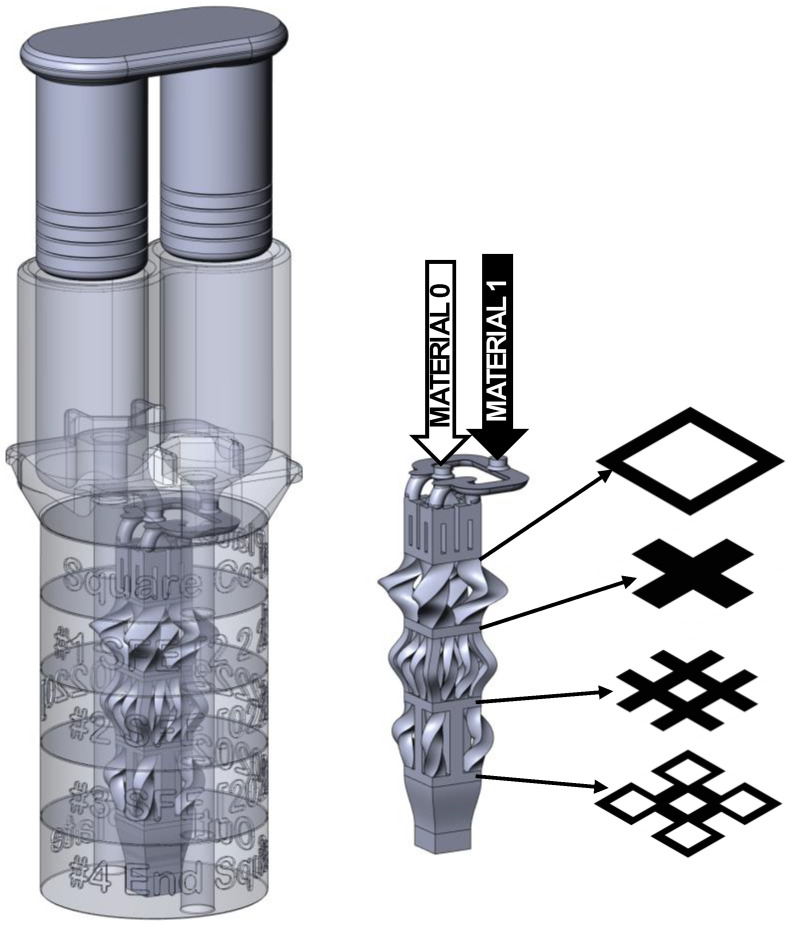
Three-dimensional models and cross-sections of the prototyped experimental design for a $G_{1}$ objective.

SFEs were also physically embodied and tested for demonstration purposes. A co-extrusion setup as shown in Figure 4 was implemented with dual injection cylinders having a bore diameter of 22 mm and a depth of 40 mm. The plungers were designed with a diametral clearance of 0.1 mm and a series of four 0.5 mm grooves to provide a dynamic seal that minimizes leakage under pressure. SFEs were designed with input and output boundaries as an 11 by 11 mm square. This section size allows for a 4 by 4 grid of flow channels each having a port geometry of 2 by 2 mm with a separating wall thickness of 1 mm. Prior work with these processes (e.g., [32]) as well as design guidelines from AM service providers indicated that the hydraulic port diameter should be greater than 2 mm with a length/diameter ratio greater than 10 to avoid clogging. In addition, a minimum wall thickness of 0.8 mm is needed between the passageways within an SFE to provide sufficient structural integrity during additive manufacturing and end use.

For the purposes of this article, the extruder and SFEs were produced in PA12 by Autotiv (Salem, NH, USA) through a selective laser sintering process having a resolution of approximately 60 microns. The implemented design for the $G_{1}$ objective is shown in Figure 4. Three-dimensional (3D) computer aided design (CAD) models for this and the $G_{2}$ designs are provided as described in the Supplemental Information. The stacked assembly was bolted together after which the bores for the inner and outer materials were, respectively, filled with 11.4 cm^3^ of blue and yellow modeling clay (Amazon part no. B000KI7XA2 and B0015ZV6CK); previous studies have successfully utilized these materials for similar flow validation purposes, demonstrating its relevance in characterizing non-Newtonian flow in extrusion processes [33,34]. The assembly was then placed in an Arbor press that extruded the clay with a force of 200 kgf on the pistons with an extrusion time of 6 s. These imposed conditions correspond to a material pressure of 2.6 MPa and a volumetric flow rate of 3.8 cm^3^/s that were adopted for flow simulation.

### 2.4. Flow Simulation

To understand the material distribution resulting from each stage of SFEs, transient multi-phase flow simulation including moving interfaces was performed with Comsol 4.6 (Stockholm, Sweden) using the level set method. The level set method [35] represents moving material interfaces using a fixed mesh by modeling the isocontour of the materials as a smooth step function between a first material represented as 0 in one domain and a second material represented as 1 in the second domain. The viscosity η for each material was modeled as isothermal but non-Newtonian with the Williamson equation [36]:

(14) $$\eta \left(\dot{\gamma }\right)=\frac{\eta_{0}}{1+{\left(\lambda \dot{\gamma }\right)}^{1-n}}$$

In this model, $\eta_{0}$ represents the Newtonian limit of the viscosity [Pa.s] at a zero-shear rate $\dot{\gamma }$ [s^−1^] and reference temperature, n is the power-law index, and λ is a characteristic relaxation time [s]. The material coefficients correspond to industrial recycled polyolefins of current interest [37]. Appendix B provides further details including the model coefficients, simulation conditions, and other details to access the CAD developed from the SFE design optimization; additional results are provided in Appendix B to investigate the roles of mesh refinement and viscosity modeling on development of the material cross-sections.

## 3. Results

### 3.1. Manually Created Designs

**Figure 5:**
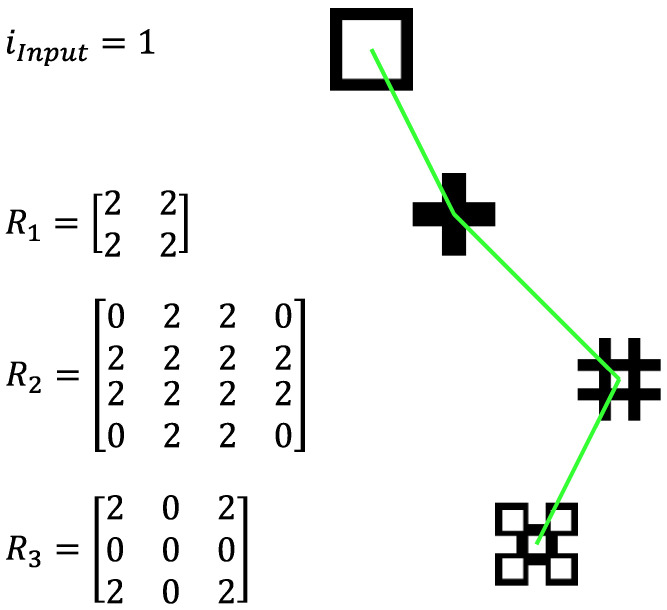
An interactively created “boxes” design specifying the input cross-section, rotation matrices having 2, 4, and 3 ports per side, and the resulting composite cross-sections.

A Matlab script, *Design_SFE*(), available as described in the Supplemental Information, was authored to interactively create architected designs for systems up to four shape forming layers. One relatively simple design, referred to as “boxes”, is shown in Figure 5 in which the first, second, and third rotation matrices, respectively, provide sets of 2 by 2, 4 by 4, and 3 by 3 ports. For demonstration purposes, selected ports are rotated by 180 degrees to convert a co-extruded square cross-section to a “plus” shape, then a “hash” shape, and finally to the “boxes” shape. The starting section $M_{0}$ is represented by an integer, *i_input_*, that maps the two starting materials to (1) a square cross-section, (2) an inverted square cross-section, (3) a bi-layer system, and (4) a tri-layer system.

### 3.2. Automated Design Solutions

Even when restricting the ports to a square geometry, the number of possible architected designs increases very quickly with the number of SFEs and number of ports in each SFE. A Matlab script, *SFE_Opt*(), was authored to implement design automation as described with respect to Figure 3 and Equation (12). To test the methods, the first objective $G_{1}$ was minimized to match the output of the automated designs with the interactively designed cross-section of Figure 5. The design vector, *x*, was developed assuming square ports so that its length of 52 elements included one material input type, three integers corresponding to the number of ports per SFE side, and three sets of 16 integers representing up to 16 port rotations per SFE. The developed script was coded and tested to be fully parallelized and also scalable with respect to the number of SFEs and ports. The material sections at each SFE level were each represented by a set of 220 by 220 pixels having values of 0 or 1; this size of 220 was selected as the product of 3, 4, 4, and 5 to provide reasonable image resolution with minimal computing resources.

**Figure 6:**
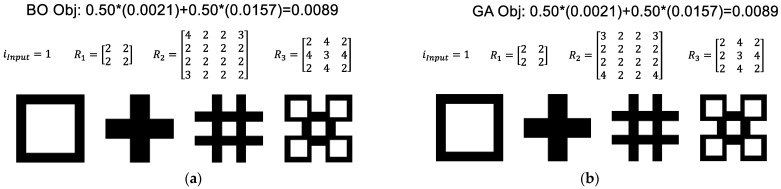
Solutions automatically generated to minimize $G_{1}$ to match the target “boxes” image through (**a**) Bayesian optimization and (**b**) genetic algorithms. Corresponding objective function values are respectively indicated.

Figure 6 provides example results for the Bayesian optimization (BO, at left) and genetic algorithms (GA, at right); the outputs are the same though the two algorithms produce different rotation matrices for the flow channels in the SFEs. The Supplemental Information provides the corresponding convergence plots for each of the solutions. Both the BO and GA methods were able to reproduce the design of Figure 5. However, the number of iterations and CPU time varied drastically. The BO called the SFE processing function only 300 times but consumed significant compute time updating its Gaussian process models and optimizing its acquisition function to determine the next point to evaluate. As a result, the BO method typically required on the order of 1200 s to converge. By comparison, the GA method performs approximately 100 times the number of function evaluations than the BO to support GA’s generative iteration. However, given that the image matrix operations are relatively trivial and speedy, the GA method was much faster, typically requiring 20 s to converge.

**Figure 7:**
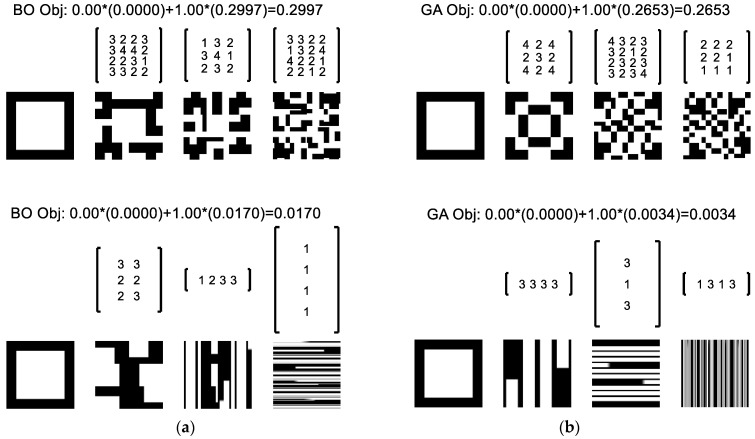
Solutions automatically generated to minimize $G_{2}$ (structural similarity index) with (**a**) Bayesian optimization and (**b**) genetic algorithms using (**top**) square and (**bottom**) rectangular ports.

Turning to the $G_{2}$ objective to minimize the structural similarity index with a white square, the same script *SFE_Opt*() was implemented with the results of Figure 7 for (top) square and (bottom) non-square ports. In the latter case, the non-square ports were modeled by augmenting the design vector, *x*, to allow the number of ports in the horizontal and vertical directions to vary such that the number of design parameters increased from 52 to 55. The modeling of non-square input and output ports was performed by mapping the partition of each input material using Matlab’s meshgrid() and then interpolating the cross-section to the output shape using Matlab’s interp2() function. This interpolation can result in non-binary material values (e.g., 0.5) depending on the size and number of the rotations as well as the size of the modeled material matrix.

Figure 7 illustrates a black and white pictorial representation of the successive architected material cross-sections resulting from the SFE design matrices provided above each section. The primary conclusion with respect to Figure 7 is that the relaxation of the square port assumption provides a significant improvement in the objective function; this result is not dissimilar to the finding of Li, Dul, and Kim [38] that material performance improved when a larger number of material microstructures is allowed in optimization. The secondary conclusion is that the GA method again outperformed the BO method for both the square and non-square problems.

**Figure 8:**
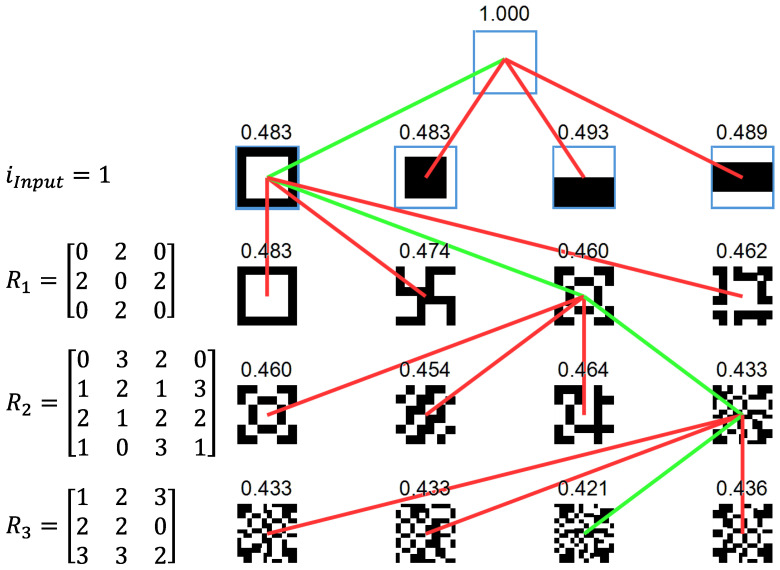
Hierarchical Bayesian optimization of the $G_{2}$ objective showing each stage of the optimization with accepted (green) design and inferior (red) solutions.

To further investigate the use of the BO method, the hierarchical implementation of Figure 2 was also implemented to minimize the $G_{2}$ objective assuming square ports. In this implementation, each of the input types is evaluated relative to the SSIM. The lowest SSIM is then adopted for further processing with the SFEs with a varying number of ports (1×1, 2×2, 3×3, and 4×4). Compared to the concurrent implementation of Figure 3, the hierarchical method greatly reduces the size of the design vector, *x*, to exactly the number of parameters needed for each optimization stage. The results are shown in Figure 8 wherein the different rows correspond to different SFE stages, the number of ports in each SFE stage is presented from 1×1 at left to 4×4  at right, and the green lines correspond to the selected solution at each step in the hierarchy. The results show that the co-extruded square and inverted square have the same $G_{2}$ relative to a white target image; this result is expected given their similar structure. The bi-layer and tri-layer have higher $G_{2}$ values so the first input type is accepted. The optimization of the first SFE tests 1 by 1, 2 by 2, 3 by 3, and 4 by 4 sets of fluid ports as depicted with the columns going from left to right. The 3 by 3 is found to provide the lowest $G_{2}$ value and so is adopted. The analysis then proceeds to the second and third SFE stages that respectively result in the selection of a 4 by 4 and then 3 by 3 stage.

In Figure 8, each SFE stage provides a reduction in the $G_{2}$ value. However, the hierarchical adoption results in a suboptimal solution relative to the concurrent selection of all design parameters, *x*, yielding the results at top in Figure 7 (BO: $G_{2}=0.300$ and GA: $G_{2}=0.265$). As previously discussed, the performance of the hierarchical solution is limited since the optimal value at an upstream stage does not guarantee a globally optimal result given the drastic changes in the architecture’s topology associated with downstream partitions and rotations. It may be that a closed-form (algebraic) solution or an inverse solution methodology (designing from the output back to the input) exists that can efficiently lead to globally optimal solutions.

### 3.3. Validation

**Figure 9:**
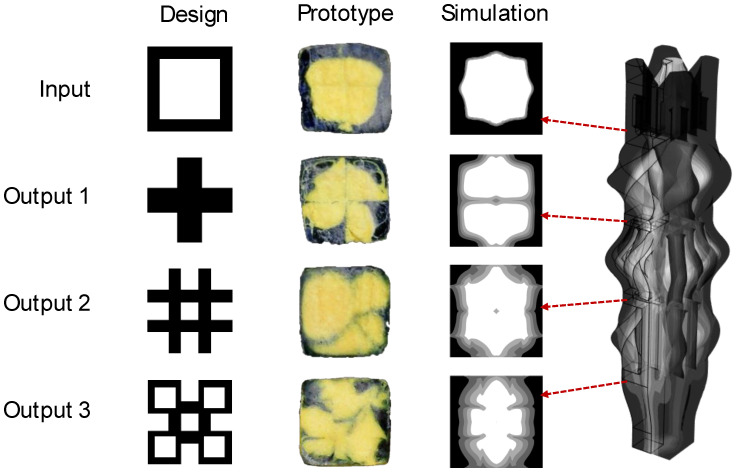
Validation results for the $G_{1}$ objective including design intent on the **left**, physical prototyping in the **center**, and multi-phase flow simulation on the **right**.

Figure 9 provides the validation results for the $G_{1}$ “boxes” objective including the design intent on the left, sections from physical prototyping in the center, and cut plots from multi-phase flow simulation on the right. The top row of Figure 9 shows that the initial condition is reasonably achieved with the physical prototype though there is some rounding as well as variation in the wall thickness of the outer material around the perimeter. By comparison, the flow simulation predicts a more octagonal shape with minor rounding of the material interfaces.

The second row corresponds to the output of the first SFE in which each flow channel in a 2 × 2 grid is rotated by 90 degrees. The physical prototype shows inconsistent results in that the flows of the top two (upper) quadrants are not significantly altered from their input state, while the bottom two (lower) quadrants clearly demonstrate some rotation in the material distribution. However, the bottom two quadrants undergo only about 45° rather than the desired 90° of rotation. Meanwhile, the flow simulation predicts a minor distribution between the center and the edges, resulting in an hourglass shape. The lack of accuracy in the flow simulation is troubling, since it precludes model-based optimization. Regardless, both the prototype and simulation results suggest that more deformation is needed to achieve the desired output; this increased deformation could be achieved by making the flow channels longer with further rotation.

The third and fourth rows of Figure 9 provide the prototyped and simulated results for the second and third SFEs, respectively. It is clear that control of the material distribution is lacking. In the prototyped material, the proportion of flow from the top-left quadrant is significantly greater than the other quadrants. This result is surprising since the flow channels in the SFEs are similarly sized and fed by fully symmetric feed channels. Regardless, the prototyped results clearly show the incursion of the outer material into the core with multiple material interfaces. By comparison, the flow simulation underpredicts the amount of flow deformation and the extent of the material interfaces observed in the physical system. Regarding gradation, the prototyped material showed little gradation between the two materials while the flow simulation suggests significant gradation as the flow progressed.

**Figure 10:**
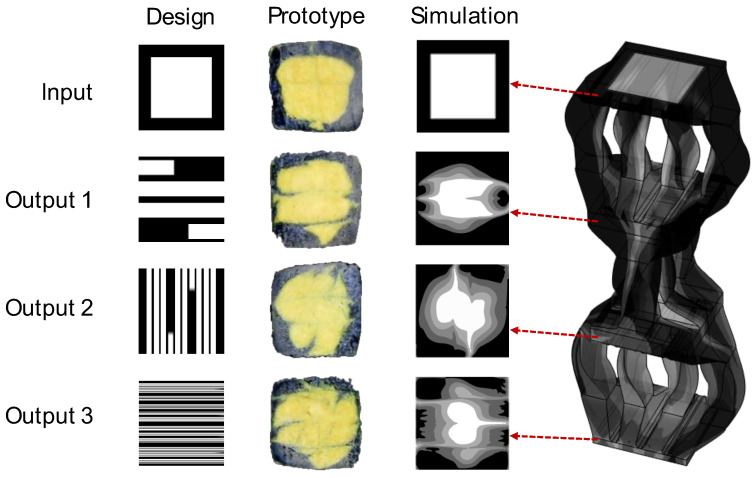
Validation results for the $G_{2}$ objective including design intent on the **left**, physical prototyping in the **center**, and multi-phase flow simulation on the **right**.

Figure 10 provides the validation results for the $G_{2}$ objective to maximize the information content of the material architecture (minimize structural similarity index to a white square) including the design intent on the left, sections from physical prototyping in the center, and cut plots from multi-phase flow simulation on the right. The nature of the results in Figure 10 is similar to those discussed for the results in Figure 9. The prototyped results again clearly show the incursion of the outer material into the core with multiple material interfaces. By comparison, the flow simulation again underpredicts the amount of flow deformation and the extent of the material interfaces observed in the physical system.

### 3.4. Structural Similarity Analysis of Resultant Sections

The developed cross-sections across different stages of the prototyped and simulated manufacturing process are compared to the intended designs using the structural similarity index (SSIM). This methodology involves preprocessing images to a uniform size of 300 × 300 pixels and converting them into grayscale to ensure consistency in comparison. The SSIM is then calculated between the images categorized into three paired comparisons: Design to Prototype (D to P), Design to Simulation (D to S), and Prototype to Simulation (P to S).

The results from this analysis for the images of Figure 9 are provided in Table 1. The SSIM values for the inlet (*i* = 0) are around 0.8 across all comparisons, indicating a reasonable translation of the design into both prototype and simulated forms. However, in the subsequent SFE stages (*i* = 1 through 3), there is a notable decline in the SSIM values. After the first SFE (*i* = 1), the SSIM values drop significantly to 0.1924 (D to P) and 0.1950 (D to S), though there is a moderate similarity between the prototype and simulation (0.4893). The similarity values are fairly stable after the second SFE stage with a slight decrease in similarity between the prototype and simulation (0.4364) compared to the first SFE stage. After the third SFE, there is a slight improvement in all SSIM values though more similarity between the prototype and simulation than with the intended design.

The structural similarity indices were also calculated for the images of Figure 10 in which the objective was to minimize the SSIM relative to white space. The results are provided in Table 2. As the material progresses through subsequent SFE stages, a marked decline is again observed in the SSIM values (0.2602, 0.3944, 0.3813 in the second set; 0.1873, 0.1958, 0.4513 in the third; and 0.0287, 0.0230, 0.3902 in the fourth), underscoring a growing discrepancy between the expected and actual results.

The consistent trend in both sets of results suggests a fundamental issue with the material processing stages not adhering to the design assumptions, leading to a decrease in predictive accuracy as the material undergoes further processing. The consistent reduction in similarity across these stages highlights the challenges in maintaining design integrity during material transformation processes. It underscores the need for refining our modeling and simulation approaches to better account for the complexities and variabilities inherent in material processing, aiming to enhance the fidelity and reliability of the outcomes at each stage of development as subsequently discussed.

## 4. Discussion

### 4.1. Optimization of Material Architecture

The use of SFEs can enable new material architectures with enhanced properties. This article described examples with objectives for (1) matching a pre-defined cross-section and (2) maximizing the negentropy relative to a uniform material. However, none of these examples claimed to be optimal, and a framework for establishing optimal architecture on an application-specific basis needs to be developed. There is significant related research such as the handling of objectives and constraints in material architectures [39,40], consideration of interfacial properties [41,42], leveraging gradient-based property distributions [43,44], utility and cost of material architectures [45,46], nonlinear material properties as a function of their processing history [47,48,49], gradation of the materials as a function of processing [50,51], and sustainability concerns [45,47], among others. Theoretically, a fundamental goal is to prove the optimality of an architected material composite for a given application. More practically, the goal is to ensure acceptable robust performance, i.e., fitness for use given uncertainty in material properties and end-use requirements [52,53]. Finite element analysis of the architected composites can be employed to provide model-based topology optimization of the stresses and structural performance.

### 4.2. Coding and Decomposition

Both the BO and GA optimizations were implemented with the design vector, *x*, of Equation (13) for square and rectangular flow ports without a shifting function. In an efficient implementation, the number of design parameters in this vector is equal to the number of ports in each SFE stage such that an SFE with a 2 by 2 set of ports would have four parameters, while an SFE with a 4 by 4 set of ports would have 16 parameters. In the current implementation, however, the design vector comprised a fixed number of parameters to accommodate the maximal number of ports. In other words, the rotation matrices for all the SFEs are sized to (the maximum number of ports per side)^2^, e.g., 4^2^ or 5^2^.

This coding has the effect of not only increasing the dimensionality of the optimization problem but also confounding the trialed changes to the dummy design parameters alongside those of the truly significant design parameters. Consider the $G_{1}$ target matching example of Figure 6. If the design parameters representing the input cross-section and number of ports per SFE stage are explicitly set as $x_{1:4}\to \left\{1,2,4,3\right\}$ and removed from the optimization problem, then the number of design parameters in the matrix *x* is reduced from 1 (for the type of input material cross-section) plus 3 (for the number of ports per side for each SFE) plus 3 × 4^2^ (for three SFEs each having up to 4 by 4 ports to rotate), which equals 52 to 2^2^ + 3^2^ + 4^2^ = 29. This change results in a 4^(52−29)^ or 7.04 $\times \;10^{13}$ reduction in the number of combinations to be explored. While the GA was robust with respect to converging to an acceptable solution with reasonable compute time, both implementations would benefit through the use of improved coding to provide the minimal number of design parameters and a more efficient design search. The number of design parameters could also be reduced through the implementation of logic supporting symmetry conditions.

Such improvements would facilitate the application of SFEs to a higher number of stages, greater number of ports per stage, shifting of ports, and other relaxations made to the current set of assumptions. More generally, however, is the need for architecture decomposition into a minimal number of stages with a minimal number of ports while also relaxing the assumptions of 1:1 mapping between the input and output ports as well as the narrow use of square or rectangular ports. While these assumptions were made to demonstrate the SFE concept and methodology, much more capable systems are envisioned with further modeling and AI advances. Badini et al. [54] provide some AI-inspired approaches that are closely related to the architectures that may be realized with SFEs.

### 4.3. Shape Forming Element Design

**Figure 11:**
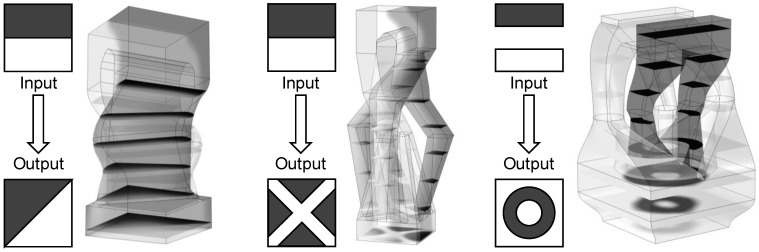
Examples of custom input-output transforms beyond square and rectangular mappings.

A square grid of input and output ports for each SFE was assumed for the initial exploration of the SFE concept. While this square port assumption was relaxed in the second example investigating the $G_{2}$ objective (minimal SSIM) with rectangular ports, the broader objective of the shape forming elements is to operate with more general transforms mapping the input to output for each SFE to create complex material architectures. The input and output ports can be designed with other shapes (triangular, circular, and non-convex), non-bijective connections (two inlets to one outlet, one inlet to two outlets, etc.), and intermediate flow channel operations to provide custom transforms between the input and output sections. Figure 11 provides some example transforms, including their preliminary flow channel designs and predicted material distributions.

The design of such custom transforms can be accomplished manually, but the work is non-trivial and tedious. The triangular transform (on the left in Figure 11) was first attempted with a simple loft connecting the rectangular input and triangular output. The resulting material distribution in the output section was highly circular and concentrated at the corners with the shortest flow path. The material distribution with the diagonal transform design was improved by implementing a first loft to an intermediate slot to distribute the flows across the hypotenuse of the triangle and then a second loft to the triangular output. The “X” design (middle of Figure 11) is the third design iteration with a routing of four black triangular legs around the white core material. The design still needs additional flow balancing to achieve a more uniform output material distribution. The “bullseye” design at right was the easiest to design and provided the desired output with three nested flow layers (innermost white circle, intermediate black annulus, and outer white annulus).

More generally, design automation of the flow geometry to create these material transforms is a critical extension that can be enabled by modeling and artificial intelligence including consideration manufacturing constraints. For a given input–output transform, the routing, cross-section shapes, and associated design parameters can be determined using the methods similar to the described BO or GA tied to flow simulation for performance prediction; each of the flow simulations shown in Figure 11 required approximately 300 s of compute time and could be readily integrated into a design system. The processing performance measures in the objective function should include at least the output material distribution and its uniformity of flow rates. Constraints related to the pressure drop, minimum flow channel dimensions, and minimum wall stock between flow channels would ensure manufacturability and processability. The minimum wall stock is critical to the creation of physically robust SFEs that can withstand the stresses imparted by the flow material during manufacturing. In practice, no failure has yet been observed in polymer or metal prototypes with a 1 mm minimum wall thickness during validation experiments. In the future, an application programming interface (API) to 3D CAD could be used to directly build the SFEs with final expert approval.

## 5. Conclusions

The concept of shape forming elements (SFEs) was described for the first time, aiming to provide a framework for the design of architected composites for industrial applications. The enablement of high-throughput manufacturing techniques for architected composites presents significant opportunities to enhance material properties through innovative material and process design strategies. Specifically, shape forming elements (SFEs) provide a systematic approach to the intelligent manufacturing of architected materials with high production rates, low cost, and a low carbon footprint.

The method was demonstrated for square and rectangular ports with a 1:1 mapping of inputs to outputs. Design automation by Bayesian optimization and genetic algorithms provided reasonable solutions for multi-objective optimization with a design vector on the order of 50 parameters. The provided results shows that the shape forming elements enable a wide array of architectures driven by the orientation and shifting of the constitutive materials within each SFE stage. Relaxation of the assumptions in the current implementation will lead to the more precise control needed to achieve more complex and capable composite architectures. While the current work demonstrated rotation, shifting, and limited shaping between rectangular ports, a more complex reshaping of input and output sections is straightforward to model but needs more advanced integration with higher level systems optimization to achieve improved material architectures. Other SFE functions such as copying and deleting would be desirable and can be computationally modeled but are difficult to practically implement in simple physical systems.

The use of shape forming elements could represent a significant step forward in the design of new material systems that combine light weight with high performance, which are increasingly demanded in industries ranging from aerospace to biomedical engineering. However, the results clearly show the need for further research to identify the optimal architected designs and methods for their manufacture.

## Figures and Tables

**Table 1:** Structural similarity indices for results of Figure 9 by image comparison.

SFE Stage	Design to Prototype	Design to Simulation	Prototype to Simulation
0 (inlet)	0.7547	0.7949	0.7875
1	0.1924	0.1950	0.4893
2	0.2002	0.1965	0.4364
3 (outlet)	0.3175	0.3456	0.4418

**Table 2:** Structural similarity indices for results of Figure 10 by image comparison.

SFE Stage	Design to Prototype	Design to Simulation	Prototype to Simulation
0 (inlet)	0.7547	0.7949	0.7875
1	0.2602	0.3944	0.3813
2	0.1873	0.1958	0.4513
3 (outlet)	0.0287	0.0230	0.3902

**Figure A1:**
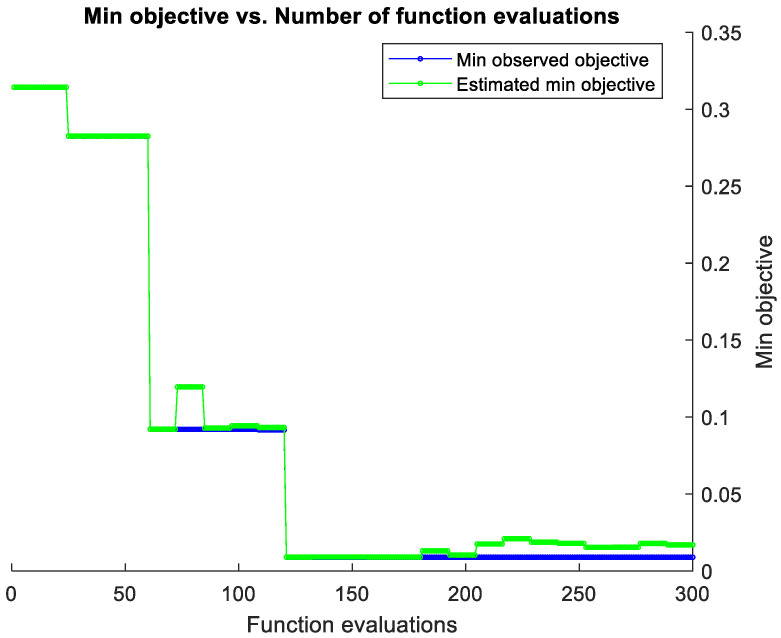
Convergence plot for converged design solution for G1 objective with Bayesian optimization (compute time: 1201 s).

**Figure A2:**
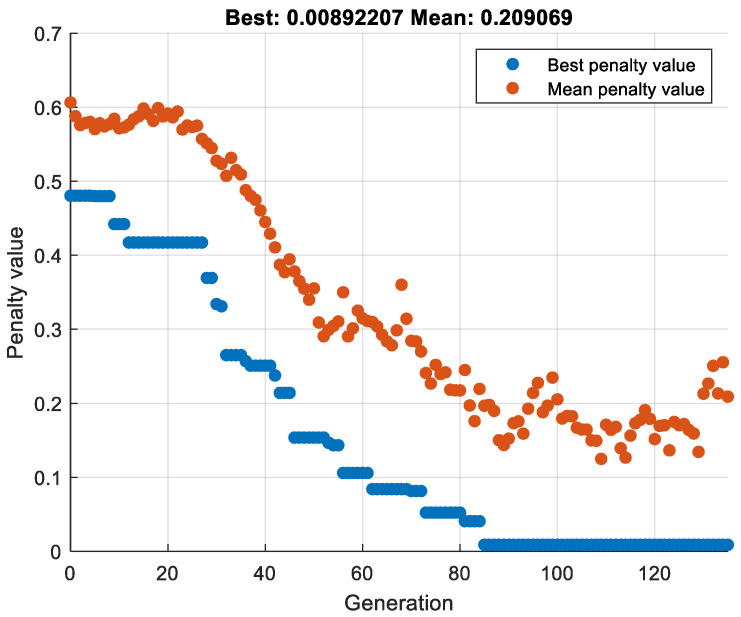
Convergence plot for converged design solution for G1 objective with genetic algorithm (compute time: 21.32 s).

**Figure A3:**
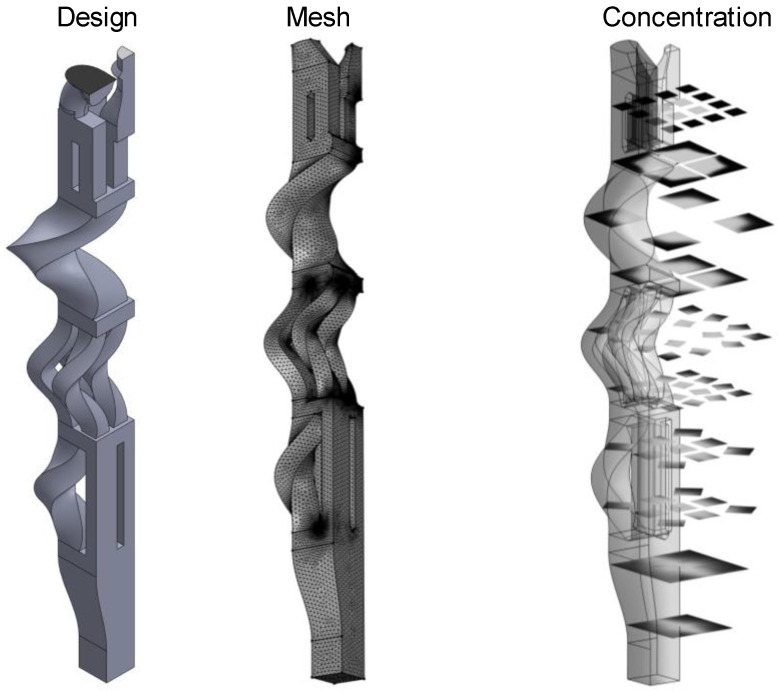
Design, mesh, and concentration results from the Comsol level-set simulation.

**Figure A4:**
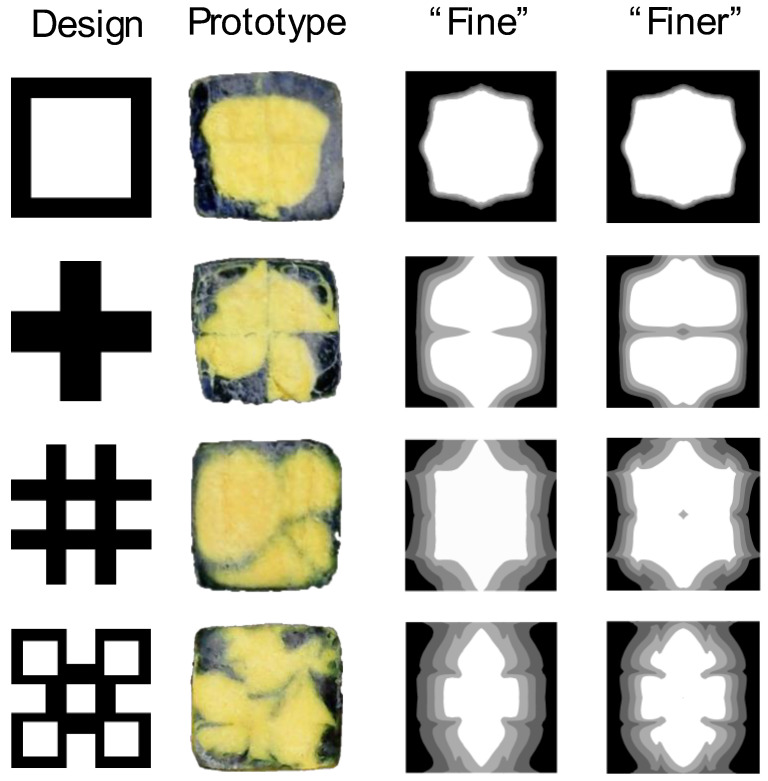
Design, prototype results, and “fine” and “finer” flow simulation results for the example of Figure 9.

**Figure A5:**
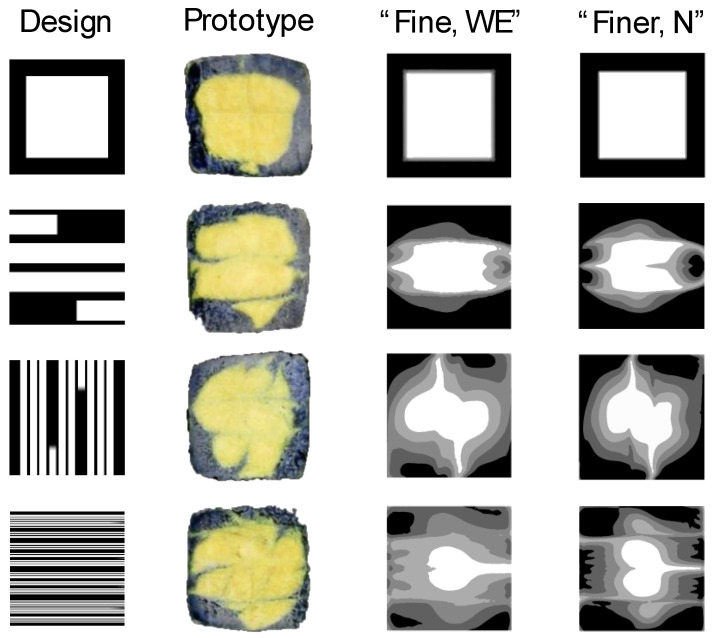
Design, prototype results, and “Fine, WE” and “Finer, N” flow simulation results for the example of Figure 10 wherein “WE” represent the non-Newtonian fluid model with the Williamson equation while “N” represents the Newtonian viscosity model.

**Figure A6:**
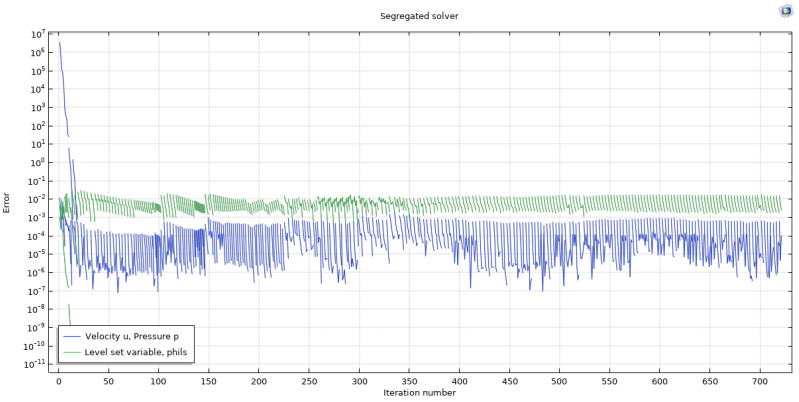
Convergence plot for Comsol flow simulation of SFE design example #2 (Figure 10).

**Figure A7:**
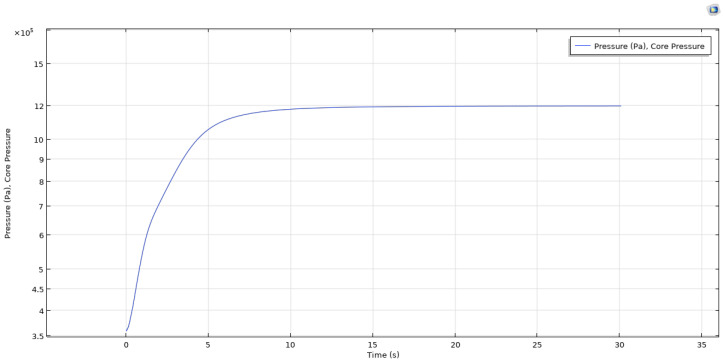
Pressure history plot for Comsol flow simulation of SFE design example #2 (Figure 10).

## Data Availability

The original contributions presented in the study are included in the article/Supplementary Materials, further inquiries can be directed to the corresponding author.

## Supplementary Materials

The following supporting information can be downloaded at https://www.mdpi.com/article/10.3390/ma17215339/s1. (1) The Matlab script Design_SFE.m to manually design shape forming elements; see the header for directions. (2) The Matlab script SFE_Opt.m to automate SFE design using Bayesian optimization or genetic algorithms providing reproduction of the presented results. There are many options and functions implemented therein to explore beyond what is described in the paper. (3) The Matlab script Compare_Results.m and accompanying images for quantitative analysis of the results. (4) The STEP files corresponding to the developed CAD supporting the *G*_1_ and *G*_2_ solutions, respectively, corresponding to the “Boxes” and low structural similarity index solutions that may be used for prototyping and simulation purposes. All information is provided as is without warranty or support. If you do use these, please reference this article.

## Appendix B. Flow Simulation Details

Transient, non-Newtonian, multi-phase flow simulations using the level set method were performed with Comsol 4.6 using a segregated solver that iteratively solves the fluid flow and level set (material concentration) fields. Materials 0 and 1 (properties indicated in Table A1) were specified at the top inlet ports with a uniform velocity corresponding to a volumetric flow rate of 5 cm^3^/s; an atmospheric pressure boundary condition was specified at the outlet. Figure A3 provides the quarter-model design, simulation, and concentration results for a quarter-symmetry model (with symmetric conditions specified for both the flow fields and material levels on the right and front planes). This first model contained 1,465,781 elements and required a solution time of 4080 s, physical memory of 8.82 GB, and virtual memory of 10.74 GB. All simulations were conducted on an AMD Ryzen 9 7900X CPU operating at 4.70 GHz with 24 logical processors.

To investigate the role of mesh dependence on the development of the developed material flow fields, a higher fidelity mesh was created by changing the Comsol mesh setting from “Fine” to “Finer.” This mesh refinement resulted in an increase in the number of tetrahedral elements to 3,703,025. The simulated processing time was also increased from 20 to 30 s to ensure a more fully developed flow field. These changes resulted in an increase in the run time to 30,195 s as well as an increase in the physical and virtual memory to 16.62 and 20.07 GB, respectively. The material concentration results for the different levels of mesh refinement are provided in Figure A4. The results suggest that the material distribution is more refined with additional mesh refinement but still far from predicting the material behavior observed via extrusion with the physical prototype.

Similar mesh refinement was also conducted for the second example of Figure 10 in which the maximum edge length of the tetrahedral elements was reduced from 0.2 to 0.1 mm. This resulted in a mesh having 6,787,935 that required 52,926 s with physical and virtual memory of 28.21 and 33.78 GB, respectively. Furthermore, to evaluate the possibility that the extruded materials are not shear thinning as modeled with the described Williamson equation, materials 0 and 1 were both modeled as Newtonian fluids with respective viscosities of 162 and 582 Pa.s. The results are shown in Figure A5 and suggest a similar refinement of the flow behavior during material processing with no additional discernible effect of the change in the viscosity model relative to the impact of mesh refinement previously demonstrated in Figure A4.

The solver convergence is shown in Figure A6 for the “Finer, N” condition with all three modeled SFEs and the die filled with material 0 initially at rest. The perturbations at iterations 100 and 150 correspond to the movement of the level set interface between materials 0 and 1 traveling through the latter SFE flow channels. The pressure history as a function of time is shown in Figure A7. The early fluctuations are associated with the travel of the level set interface through the flow channels in the SFEs with the propagation of material 1 that has higher viscosity (582 Pa·s) than the material 0 (162 Pa·s). The pressure plateau after 15 s suggests that the material interfaces depicted in Figure 10 are fully developed as simulated.

**Table A1:** Flow simulation material data for materials 0 and 1.

Material Property	Material 0	Material 1
MFR (g/10 min, 2.16 kg)	8.63 ± 0.05	5.67 ± 0.12
Melt density, kg/m^3^	850	850
Willamson, zero-shear viscosity, η_0_(230 °C) [Pa]	1127	1935
Willamson, power-law index, *n*	0.249	0.283
Willamson, characteristic relaxation time, λ [s]	0.0232	0.0437

## Appendix C. Description of Matlab Functions

Bayesopt: The bayesopt function in Matlab is a statistical technique for optimizing nonlinear objective functions that are expensive to evaluate. It operates by constructing a probabilistic model of the objective function and iteratively selecting the most promising data points to evaluate based on current estimates. The function integrates prior belief about the objective function and updates the model as new data are gathered, balancing exploration of the design space with exploitation of known good regions. This approach is particularly advantageous for optimizing complex material architectures where the direct evaluation of every design configuration is computationally prohibitive.

Compare_Results: The Compare_Results.m Matlab script was written for this article to compare the images from various stages of material processing experiments, specifically for Design (D), Prototype (P), and Simulation (S). This script automates the loading, processing, and comparison of images corresponding to these stages using the structural similarity index (SSIM) to quantitatively assess their similarity. The script can handle both grayscale and black/white images with a Boolean option (bGray) to toggle between these modes based on the analysis needs; each image is resized to a standard dimension (300 × 300 pixels) to ensure consistency in comparison.

Design_SFE: The Design_SFE.m function is a Matlab script authored in this research that is designed to create and visualize shape forming elements (SFEs) with configurable square port rotations for material distribution in architected composites. The function includes a graphical user interface (GUI) that allows users to interactively set the number of ports per stage and adjust the rotation of each port using keyboard inputs. The script initializes a figure with controls to navigate through stages and modify port configurations, supporting the design of complex flow channels in multi-layered structures. This tool is built to aid in the visualization and manual adjustment of SFE configurations, enabling a hands-on approach to designing optimized material architectures for various manufacturing processes.

Ga: The ga function in Matlab implements genetic algorithms (GAs), which is a class of optimization techniques inspired by natural selection that are effective for solving complex optimization problems with large solution spaces. This function works by generating a population of candidate solutions, which are then subjected to operations such as selection, crossover, and mutation to evolve the population toward better solutions over successive generations. Each candidate is evaluated based on a fitness function, and the best-performing individuals are more likely to contribute to the next generation. Genetic algorithms are particularly useful for exploring high-dimensional design spaces and finding global optima in problems where traditional gradient-based optimization methods might fail due to nonlinearity or discontinuities.

Immse: The immse function in Matlab is an acronym for IMage Mean Squared Error, which is used to compute the mean squared error between two images. This function is typically employed to quantify the difference between a reference image and a test image, providing a measure of the distortion or error introduced during image processing or transmission. The mean squared error calculated by immse reflects the average of the squares of the pixel intensity differences between corresponding pixels of the two images. It is a critical tool in image analysis, and it is particularly useful in optimization routines for image processing where reducing the error metric is a common objective, such as in the manuscript’s context for validating composite architectures.

Interp2: The interp2 function in Matlab performs two-dimensional interpolation on matrices, which is used for estimating intermediate values between discrete data points in a grid. This function can accept various methods of interpolation such as linear (used in this research), cubic, or spline, allowing users to choose the one that best suits their application needs. In this application, it supports the creation of smooth representations of the material concentrations in matrices of varied sizes and aspect ratios as discussed in the context of transforming material cross-sections in the manuscript.

Meshgrid: The meshgrid function in Matlab is used to create two 2D arrays containing the coordinates of points on a grid, where one array holds the x-coordinates and the other holds the y-coordinates. It is a method for mapping functions over a grid, enabling vectorized operations on matrices that represent points across a meshed domain. This function is used to support the interpolation of material distributions when mapping material concentrations in the partition of the output from one SFE to an input partition of the next SFE and they have different pixel sizes.

Rot90: The rot90 function in Matlab rotates a matrix or an array by 90 degrees counterclockwise. This function is used to manipulate image data or reorient matrices to align with different coordinate systems or perspectives. A second argument specifies the number of times the matrix should be rotated by 90 degrees, enabling flexibility in achieving the desired orientation. For example, the function call rot90(A,2) would rotate the matrix A by 180 degrees.

SFE_Opt: The SFE_Opt.m function is a Matlab script authored for this research that optimizes shape forming elements (SFEs) through integer-valued Bayesian optimization (BO) and genetic algorithms (GAs). This script is designed to optimize the design of SFEs used in creating architected material composites by adjusting parameters such as port shapes, sizes, and rotations to achieve specific performance objectives. Users can configure the optimization process through Boolean options to choose between using Bayesian optimization or genetic algorithms, specify the shape of ports (square or non-square), and decide on varying input types which directly affect the degrees of freedom in the optimization problem. Key options in the script allow users to penalize the number of ports, append results to an archive for later review, and display cross-sectional views and objective values during optimization runs. The script also accommodates non-rectangular ports and includes placeholders for future functionalities like solving from the bottom up or sequentially through stages. Different objectives functions are also implemented including placeholders and trial functions related to mechanical properties.

SSIM: The ssim function in Matlab calculates the structural similarity index (SSIM) between two images, which is a method for measuring the similarity between two images. SSIM is a perception-based model that considers image degradation as a perceived change in structural information, incorporating aspects of luminance, contrast, and structure. The SSIM function returns a decimal value between 0 and 1 that, respectively, indicates no and perfect similarity.
