# Supplementary figures and images for: Design of Shape Forming Elements for Architected Composites via Bayesian Optimization and Genetic Algorithms: A Concept Evaluation
Source: Materials (Basel). 2024 Oct 31;17(21):5339. doi: 10.3390/ma17215339 (PMC11547659; doi:10.3390/ma17215339)

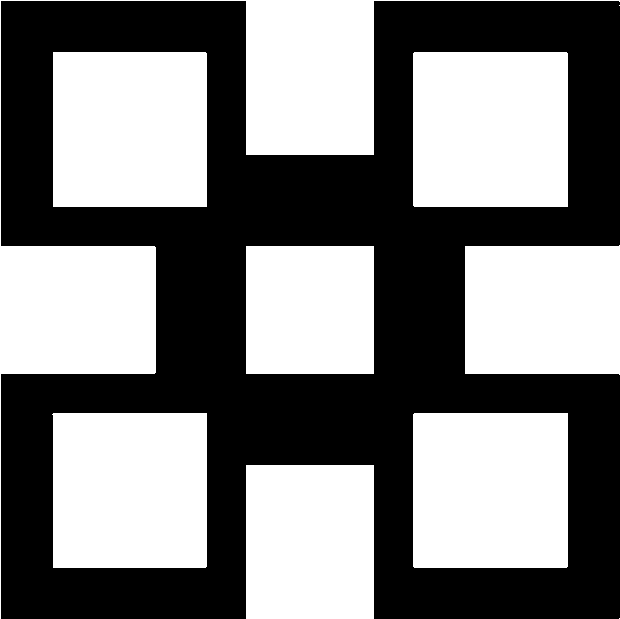

Supplement: Supplementary file 1 [file materials-17-05339-s001.zip › Boxes.bmp]

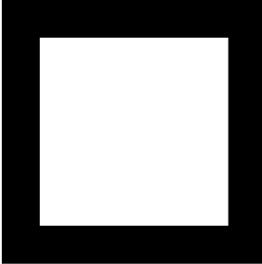

Supplement: Supplementary file 1 [file materials-17-05339-s001.zip › SSIM Results Analysis/EX1-0-D.jpg]

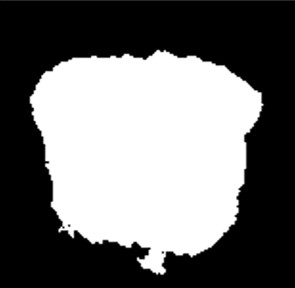

Supplement: Supplementary file 1 [file materials-17-05339-s001.zip › SSIM Results Analysis/EX1-0-P.jpg]

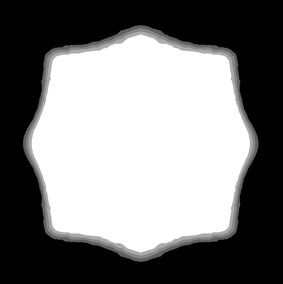

Supplement: Supplementary file 1 [file materials-17-05339-s001.zip › SSIM Results Analysis/EX1-0-S.jpg]

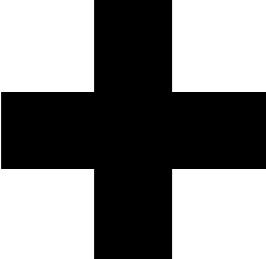

Supplement: Supplementary file 1 [file materials-17-05339-s001.zip › SSIM Results Analysis/EX1-1-D.jpg]

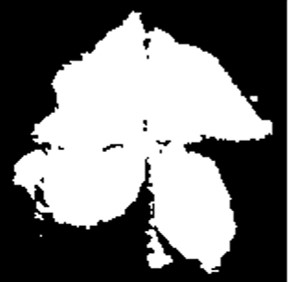

Supplement: Supplementary file 1 [file materials-17-05339-s001.zip › SSIM Results Analysis/EX1-1-P.jpg]

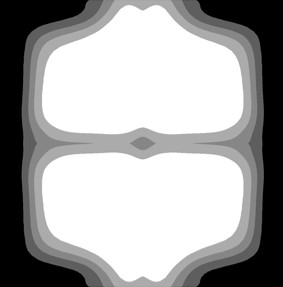

Supplement: Supplementary file 1 [file materials-17-05339-s001.zip › SSIM Results Analysis/EX1-1-S.jpg]

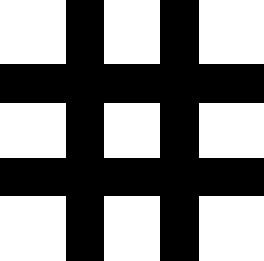

Supplement: Supplementary file 1 [file materials-17-05339-s001.zip › SSIM Results Analysis/EX1-2-D.jpg]

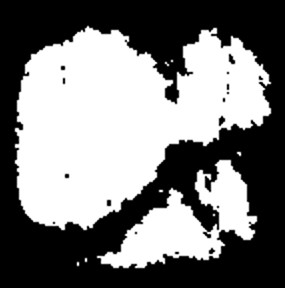

Supplement: Supplementary file 1 [file materials-17-05339-s001.zip › SSIM Results Analysis/EX1-2-P.jpg]

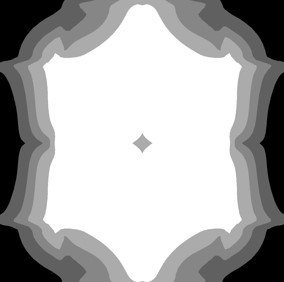

Supplement: Supplementary file 1 [file materials-17-05339-s001.zip › SSIM Results Analysis/EX1-2-S.jpg]

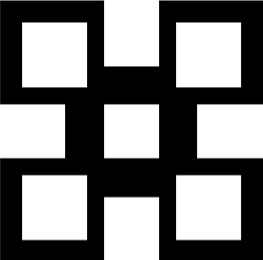

Supplement: Supplementary file 1 [file materials-17-05339-s001.zip › SSIM Results Analysis/EX1-3-D.jpg]

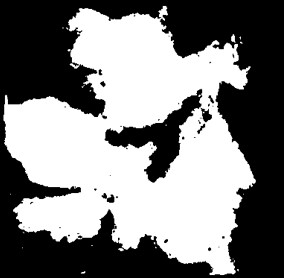

Supplement: Supplementary file 1 [file materials-17-05339-s001.zip › SSIM Results Analysis/EX1-3-P.jpg]

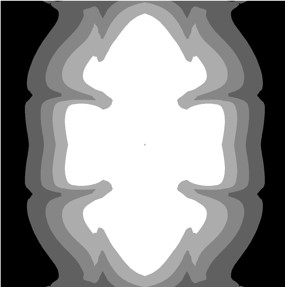

Supplement: Supplementary file 1 [file materials-17-05339-s001.zip › SSIM Results Analysis/EX1-3-S.jpg]

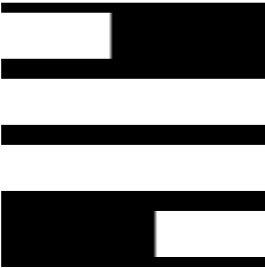

Supplement: Supplementary file 1 [file materials-17-05339-s001.zip › SSIM Results Analysis/EX2-1-D.jpg]

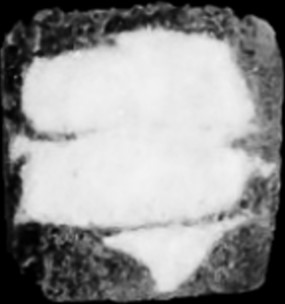

Supplement: Supplementary file 1 [file materials-17-05339-s001.zip › SSIM Results Analysis/EX2-1-P.jpg]

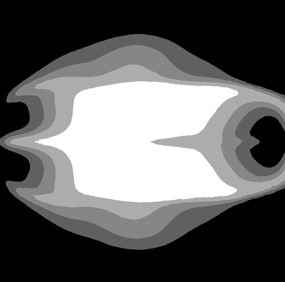

Supplement: Supplementary file 1 [file materials-17-05339-s001.zip › SSIM Results Analysis/EX2-1-S.jpg]

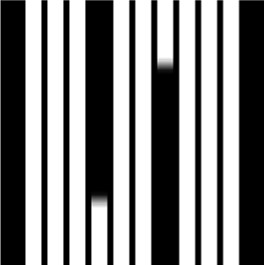

Supplement: Supplementary file 1 [file materials-17-05339-s001.zip › SSIM Results Analysis/EX2-2-D.jpg]

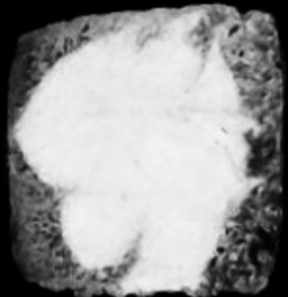

Supplement: Supplementary file 1 [file materials-17-05339-s001.zip › SSIM Results Analysis/EX2-2-P.jpg]

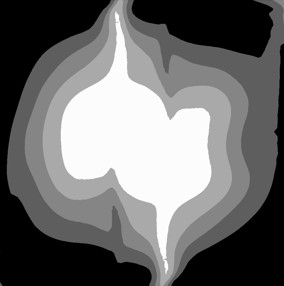

Supplement: Supplementary file 1 [file materials-17-05339-s001.zip › SSIM Results Analysis/EX2-2-S.jpg]

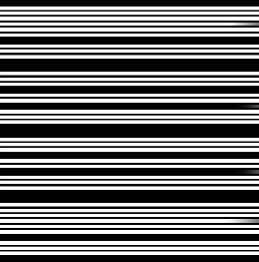

Supplement: Supplementary file 1 [file materials-17-05339-s001.zip › SSIM Results Analysis/EX2-3-D.jpg]

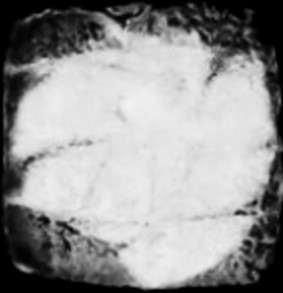

Supplement: Supplementary file 1 [file materials-17-05339-s001.zip › SSIM Results Analysis/EX2-3-P.jpg]

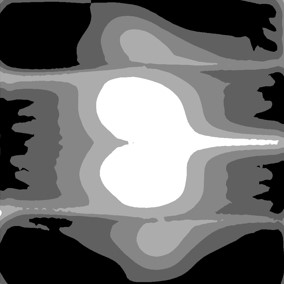

Supplement: Supplementary file 1 [file materials-17-05339-s001.zip › SSIM Results Analysis/EX2-3-S.jpg]
